# Psychometric properties of the Chinese version of the Arm Activity Measure in people with chronic stroke

**DOI:** 10.3389/fneur.2023.1248589

**Published:** 2023-09-22

**Authors:** Nga Huen Chan, Shamay S. M. Ng

**Affiliations:** ^1^Department of Rehabilitation Sciences, The Hong Kong Polytechnic University, Hong Kong, Hong Kong SAR, China; ^2^Research Centre for Chinese Medicine Innovation, The Hong Kong Polytechnic University, Hong Kong, Hong Kong SAR, China

**Keywords:** stroke, rehabilitation, psychometric properties, upper limb, Arm Activity Measure

## Abstract

**Introduction:**

The Arm Activity Measure was developed to assess active and passive functions of the upper limb in people with unilateral paresis, but a Chinese version is not available and its psychometric properties have not been specifically tested in people with stroke. This study aimed to translate and culturally adapt the Chinese version of the Arm Activity Measure (ArmA-C) and establish its psychometric properties in people with chronic stroke.

**Methods:**

The psychometric properties of ArmA-C were determined in 100 people with chronic stroke.

**Results:**

The ArmA-C had good test–retest reliability (intraclass correlation coefficients [ICC] = 0.87–0.93; quadratic weighted Kappa coefficients = 0.53–1.00). A floor effect was identified in section A of the ArmA-C. The content validity and internal consistency (Cronbach's alpha coefficients = 0.75–0.95) were good. The construct validity of the ArmA-C was supported by acceptable fit to the two-factor structure model and significant correlations with the Fugl-Meyer Assessment for Upper Extremity score, grip strength, the Wolf Motor Function Test score, the Trail Walking Test completion time, and the Oxford Participation and Activities Questionnaire scores.

**Conclusions:**

The ArmA-C is reliable and valid for assessing active and passive functions in people with chronic stroke.

## 1. Introduction

Upper-limb motor impairment is common after a stroke and has a great influence on the functional performance of people with stroke. It has been shown to have a high prevalence rate of 77% in people with subacute stroke (1), and few of those affected achieve complete functional recovery after the subacute stage. More than 50% of people with chronic stroke suffer from persistent motor impairments of the upper limb, such as spasticity, weakness, and abnormal muscle synergy (2), on the affected side (3). Deficits in both the proximal and distal segments of the upper limb may contribute to functional loss, as impairments in the proximal arm may cause difficulties in transporting and rotating the hand and impairments in the distal arm may reduce the ability to contact and interact with objects (4). Upper-limb function greatly influences the performance of activities of daily living (*r* = 0.74, *p* < 0.001) (5) and the social participation (β = 0.14, *p* = 0.05) of people with stroke (6). Loss of upper-limb function may cause restrictions in daily activities and social engagement (7). Moreover, upper-limb function is also related to walking performance after stroke (β = 0.11, *p* = 0.047) due to the role of arm swing in maintaining postural balance and controlling the center of mass movement (8, 9).

Impairments in the active and passive functions of the upper limb affect the functional recovery and independence of people with stroke. Active and passive functions are two separate concepts indicating different functional aspects in people with stroke (10). The goals for upper-limb motor rehabilitation are to improve performance in holding and manipulating objects, defined as active function, and the ability to care for the affected arm, defined as passive function (11). An instrument with both active and passive function constructs may help clinicians and researchers to comprehensively assess the functional performance of the affected upper limb to improve clinical practice and evaluate the effectiveness of interventions used in stroke rehabilitation.

Several self-reported instruments have been developed to reflect the “real-life” active and passive functions of the upper limb in people after stroke or brain injury. For example, the Leeds Adult Spasticity Impact Scale (LASIS) measures the perceived difficulty in passive function tasks, such as “cleaning the palm of the affected hand”, and low-level active function tasks, such as “difficulty balancing in standing,” to evaluate upper-limb performance in real-life contexts, but it has not undergone psychometric testing (12). Similarly, the ABILHAND questionnaire assesses upper-limb performance by examining the perceived ease or difficulty in performing daily tasks using the upper limb. However, it mainly focuses on active function, and there are few items assessing passive function (13). The floor effect may also exist in people with stroke due to the complexity of the items in the ABILHAND, such as “hammering a nail” and “threading a needle” (11, 14).

To address the inadequacies of LASIS and ABILHAND, the Arm Activity Measure (ArmA) was developed to measure upper-limb function in people with unilateral paresis. It assesses the subjective difficulty in performing active and passive functional aspects of daily activity tasks. Section A of the ArmA includes eight items that measure the ability to perform passive functional tasks, such as maintaining hygiene and dressing. Section B includes 13 items that examine the ability to perform active functional tasks with the affected hand, such as holding and manipulating objects. The original English version of the ArmA has demonstrated good internal consistency and test–retest reliability in people with upper-limb paresis due to stroke, brain injury, and other neurological conditions (15). It has also been shown that scores for the original English version of the ArmA significantly correlate with LASIS (*r*_*s*_ = 0.48–0.50) and the Disabilities of Arm Shoulder and Hand (*r*_*s*_ = 0.63) scores (15). The ArmA has previously been translated into Thai (16) and Swedish (17).

Although the ArmA has potential as a self-reported questionnaire that equally covers and assesses both the active and passive functions of people with stroke, it has not been translated into Chinese, and its psychometric properties have not been systematically assessed in community-dwelling people with chronic stroke. The objectives of this study were to (1) translate and culturally adapt the original English version of the ArmA into Chinese and (2) to examine the test–retest reliability, ceiling and floor effects, content validity, internal consistency, and construct validity of the Chinese version of the ArmA in people with stroke and its correlations with other stroke-specific function-related outcome measures.

## 2. Materials and methods

### 2.1. Participants and sample size calculation

A convenience sample of people with stroke was recruited from February to June 2021 from local self-help groups in Hong Kong. People with stroke were included in the study if they: (1) were 50 years old or above; (2) were diagnosed with stroke at least 12 months previously; (3) scored 7 or more on the Abbreviated Mental Test (18); (4) were community-dwelling; and (5) were able to understand Cantonese. Participants were excluded if they had another neurological condition, a musculoskeletal disorder, or any unstable medical condition that could affect their assessment.

Written informed consent was obtained from all participants before their enrolment in the study. The study was approved by the Human Subjects Ethics Committee of The Hong Kong Polytechnic University and was conducted in accordance with the guidelines of the Declaration of Helsinki.

At least 100 people with stroke were required because factor analysis was involved in this study (19). In previous studies, the test–retest reliability of different versions of the ArmA was evaluated using the quadratic weighted Kappa coefficient. The lowest weighted Kappa coefficient reported in prior studies is 0.83 (17). Our sample size calculation showed that 36 participants would be required to establish sufficient test–retest reliability at a power of 0.8 and a significance level of 0.05. Thus, 100 people with stroke were eventually recruited, and 36 participants were invited to complete a reassessment after 1 week.

### 2.2. Translation and cultural adaption of the ArmA

The ArmA was translated based on the recommendations of Beaton et al. (20) after obtaining permission from the developers of ArmA. The English version of the ArmA was independently translated into Chinese by two bilingual translators. One of the translators has a rehabilitation training background, and the other one is a professional translator without a rehabilitation training background. The translators reviewed two forward translations and resolved any discrepancies by discussion to reach a consensus and produce a reconciled translated version. The reconciled version was then back-translated into English by another two independent translators who were not involved in forward translation, including one with a rehabilitation training background and a professional translator without a rehabilitation training background. These two translators compared the back-translated versions with the original English version and identified and resolved any conceptual discrepancies by discussion. The reconciled translation was then adopted as the initial Chinese version of the ArmA.

The semantic, experiential, conceptual, and idiomatic equivalence of the Chinese version of the ArmA were evaluated by a panel of five professionals, including physiotherapists, nurses, and a professional translator. Each item was examined using a 4-point Likert scale, with 1 representing “not relevant,” 2 representing “somewhat relevant,” 3 representing “quite relevant,” and 4 representing “highly relevant”. The initial Chinese version of the ArmA was tested on nine people with stroke to ascertain its fluency, clarity, and comprehensibility. The final Chinese version of the ArmA (ArmA-C) was produced and used in the current study.

### 2.3. Outcome measures

#### 2.3.1. ArmA

The ArmA-C is a self-reported questionnaire to assess passive (section A) and active (section B) real-life arm function (21). Section A comprises eight items that assess the difficulty in caring for the affected limb, while section B comprises 13 items that examine the difficulty in performing tasks or activities using the affected arm (21). Each item is scored using a 5-point Likert scale, where 0 indicates no difficulty, 1 indicates mild difficulty, 2 indicates moderate difficulty, 3 indicates severe difficulty, and 4 indicates the inability to perform the activity. The total scores range from 0 to 32 for section A and 0 to 52 for section B. Lower scores represent less perceived difficulty in functional tasks and better arm function on the affected side. The original English version of the ArmA has been shown to be valid (Cronbach's alpha = 0.85–0.96) and reliable (quadratic weighted Kappa coefficients = 0.90–0.93) in people with upper-limb paresis due to stroke, brain injury, and other neurological conditions (15).

#### 2.3.2. Fugl–meyer assessment for upper extremity

The motor domain of Fugl–Meyer Assessment for Upper Extremity (FMA-UE) measures reflex activities, movements, and coordination of the affected upper limb (22). The items in FMA-UE, except two items assessing the reflex activity, are rated with 0, 1, or 2 points, which represent “cannot perform,” “performs partially,” or “performs fully,” respectively. The total score ranges from 0 to 66, with higher scores representing better motor performance of the upper limb. FMA-UE scores of 0 to 31 indicate severe impairment, and scores of 32 to 66 indicate mild-to-moderate impairment in the upper extremity (23). The inter-rater reliability of the FMA-UE has been found to be excellent [intraclass correlation coefficient (ICC) = 0.98] in people with stroke (24).

#### 2.3.3. Grip strength

The grip strength of the affected hand was measured using a digital hand dynamometer (EH101; Zhongshan Canry Electronic Co. Ltd., Zhongshan, China). The participant was seated in a comfortable chair, with shoulder adduction at 0° and elbow flexion at 90°. They were asked to grip the hand dynamometer with maximal isometric effort for at least 5 s. No other body movements were allowed. Two trials were performed by each participant with at least a 2-min rest between trials to avoid muscle fatigue and the mean strength was calculated. Excellent test–retest reliability (ICC = 0.97–0.98) of grip strength measurements has been shown in healthy adults using this hand dynamometer (25).

#### 2.3.4. Wolf motor function test

The Wolf Motor Function Test (WMFT) evaluates the motor abilities of the affected and unaffected upper extremities using six timed joint-segment movement items and nine timed functional items (26). The quality of movement of both arms is rated on a 6-point scale ranging from 0 (no attempt made) to 5 (movement appears to be normal), with total scores ranging from 0 to 75 (27). A maximum time of 120 s is allowed for the completion of each timed task (27). A higher score in the WMFT indicates better movement capability of the upper extremities. The WMFT has demonstrated excellent inter-rater reliability (ICC = 0.88–0.97) and test–retest reliability (*r* = 0.90–0.95) in people with chronic stroke (28).

#### 2.3.5. Trail walking test

The Trail Walking Test (TWT) simultaneously measures motor (locomotion and turning) and cognitive functions (visual search function and short-term memory) (29). In the TWT, participants are required to walk to 15 numbered cones sequentially at their normal walking speed (30). One trial was performed by each participant, and the completion time was recorded. Good test–retest reliability (ICC = 0.88) and excellent inter-rater reliability (ICC = 0.99) have been found for the TWT in people with chronic stroke (30).

#### 2.3.6. Oxford participation and activity questionnaire

The Chinese version of the Oxford Participation and Activities Questionnaire (Ox-PAQ-C) consists of 23 items that assess the level of participation and activity based on three domains, namely routine activities, social engagement, and emotional wellbeing (31). The items are scored according to a 5-point Likert scale (0 = never; 1 = rarely; 2 = sometimes; 3 = often; 4 = always). The total scores for the routine activities, social engagement, and emotional wellbeing subscales are 56, 16, and 20, respectively. Higher scores in the Ox-PAQ-C represent greater difficulties in participation and activity. The Ox-PAQ-C has demonstrated good internal consistency (Cronbach's alpha = 0.86–0.91) and excellent test–retest reliability (ICC = 0.91–0.94) in people with chronic stroke (32).

### 2.4. Statistical analysis

The item-level content validity index (I-CVI) value was calculated using the number of panel members giving a relevance rating of 3 or 4 for the item, divided by the total number of panel members. Items with an I-CVI value equal to or higher than 0.78 are considered to have good content validity (33). The scale-level content validity index (S-CVI) was calculated by averaging the I-CVI values of all items. An S-CVI value of 0.90 or above indicates acceptable content validity for the whole scale (33).

Statistical analyses were performed using the Statistical Package for the Social Sciences (version 28.0; IBM Corp, Armonk, NY, USA). The demographic characteristics of people with stroke were summarized using descriptive statistics. The scores in the section A and B of ArmA-C obtained by participants with different characteristics were compared using an independent Student's t-test, which can compare the continuous variables from two different categories. The test–retest reliability of the two subscales between 2 days were evaluated using ICC_3, 1_, which is the ICC model with two-way mixed effects by absolute agreement. ICC values <0.50, 0.50 to 0.75, 0.75 to 0.90, and >0.90 indicate poor, moderate, good, and excellent reliability, respectively (34). Item-level test–retest reliability was also assessed using the quadratic weighted Kappa coefficient. Weighted Kappa coefficients of 0.01–0.20, 0.21–0.40, 0.41–0.60, 0.61–0.80, and 0.81–1.00 reflect slight, fair, moderate, substantial, and almost perfect agreement, respectively (35). To examine the ceiling or floor effects of the ArmA-C, the proportions of participants obtaining the maximum and minimum scores were calculated. A ceiling or floor effect of <10% is considered to be acceptable (36).

The internal consistency of the ArmA-C was assessed using Cronbach's alpha coefficient. A Cronbach's alpha coefficient >0.75 is indicative of good internal consistency (37). To assess construct validity, confirmatory factor analysis with the two subscales in the original ArmA (15) was performed to test the theoretical foundation in ArmA-C. The fit of data to the model was assessed using the following criteria: the ratio of chi-square to degree of freedom (χ^2^/df) < 3.0, the comparative fit index (CFI) > 0.90, and the root mean square error approximation (RMSEA) < 0.06 (38). In addition, the construct validity of ArmA-C was investigated by hypothesis testing. We hypothesized strong to very strong correlations between ArmA-C scores and FMA score, grip strength, and WMFT scores, as they examine upper limb motor function. Moreover, a weak to moderate correlation was expected between ArmA-C scores and TWT completion time, because the upper limb motor function may be associated with the performance in gait control of people with stroke (8, 9). We also hypothesized a weak to moderate correlation between ArmA-C scores and Ox-PAQ-C scores, as the motor function has a dynamic interaction with activity and participation of people with stroke based on the framework of International Classification of Functioning, Disability and Health. The degree of correlation was determined using Spearman's correlation coefficient (*r*_*s*_). The strength of the correlation was defined as weak (*r*_*s*_ = 0.20–0.29), moderate (*r*_*s*_ = 0.30–0.39), strong (*r*_*s*_ = 0.40–0.69), or very strong (*r*_*s*_ ≥ 0.70) (39).

## 3. Results

### 3.1. Characteristics of the participants

The characteristics of the participants recruited in this study are described in Table 1. One hundred people with chronic stroke were recruited. The median scores for sections A and B of the ArmA-C were 1.00 (interquartile range [IQR] = 5.00) and 28.50 (IQR = 33.00), respectively.

**Table 1:** Demographic characteristics of the participants (*n* = 100).

**Characteristics**	
Age (Mean ± SD)	65.00 ± 6.18
Years since stroke (Mean ± SD)	7.76 ± 4.44
**Gender, number (%)**	
Male	58 (58.00)
Female	42 (42.00)
**Employment status, number (%)**	
Employed	3 (3.00)
Unemployed/retired	97 (97.00)
**Cause of stroke, number (%)**	
Ischemic	68 (68.00)
Hemorrhagic	32 (32.00)
**Dominant-side hemiplegia, number (%)**	
Yes	53 (53.00)
No	47 (47.00)
**Upper extremity impairment level, number (%)**	
Mild-to-moderate	75 (75.76)
Severe	24 (24.24)

SD, standard deviation.

Table 2 shows a comparison of the ArmA-C scores for participants with various characteristics. Significant differences were found between participants with different upper-extremity impairment levels, as determined by the FMA-UE score. Stroke participants with a severe impairment level (FMA-UE scores of 0–31) (23) had significantly higher ArmA-C scores in both sections A and B than those with a mild-to-moderate impairment level (FMA-UE scores of 32–66) (23). There were no significant differences in ArmA-C scores according to sex, employment status, cause of stroke, or the presence of dominant-side hemiplegia.

**Table 2:** Comparisons for the scores of Arm Activity Measure of stroke survivors with different characteristics.

**Characteristics**	**Section A (Mean ±SD)**	***t*-value (*p*-value)**	**Section B (Mean ±SD)**	***t*-value (*p*-value)**
Gender		t = 1.68 (*p* = 0.096)		t = 0.67 (*p* = 0.505)
Male	3.33 ± 3.65		26.28 ± 16.20	
Female	2.14 ± 3.23		24.12 ± 15.51	
Employment status		t = −1.26 (*p* = 0.212)		t = 1.38 (*p* = 0.172)
Employed	5.33 ± 9.24		13.00 ± 14.93	
Unemployed/retired	2.75 ± 3.28		25.75 ± 15.82	
Cause of stroke		t = −0.58 (*p* = 0.567)		t = −1.02 (*p* = 0.312)
Ischemic	2.69 ± 3.54		24.26 ± 16.06	
Hemorrhagic	3.13 ± 3.50		27.72 ± 15.45	
Dominant-side hemiplegia		t = −1.67 (*p* = 0.099)		t = −1.98 (*p* = 0.050)
Yes	2.28 ± 3.43		22.45 ± 15.96	
No	3.45 ± 3.54		28.66 ± 15.28	
Upper extremity impairment level		t = 2.64 (*p* = 0.010)		t = 6.99 (*p* < 0.001)
Mild-to-moderate	2.35 ± 3.36		20.12 ± 14.74	
Severe	4.46 ± 3.61		41.54 ± 4.67	

SD, standard deviation.

### 3.2. Reliability

Test–retest reliability evaluations of the ArmA-C resulted in ICC values of 0.87 and 0.93 for sections A and B, respectively. The quadratic weighted Kappa coefficients of all items ranged from 0.53 to 1.00 (Table 3). There were no ceiling effects in section A. However, 45 participants (45%) achieved the lowest score in section A, indicating a floor effect. There were no ceiling or floor effects identified in section B.

**Table 3:** Test-retest reliability of the Chinese version of the Arm Activity Measure.

**Item**	**Weighted Kappa value**	**95% confidence interval (lower; upper)**
**Section A**		
1. Cleaning the palm of the hand	0.72	0.47; 0.97
2. Cutting finger nails	0.85	0.72; 0.99
3. Cleaning the armpit	0.78	0.58; 0.99
4. Cleaning the elbow crease	0.78	0.57; 0.99
5. Positioning arm on a cushion or support in sitting	0.74	0.50; 0.97
6. Putting arm through a garment sleeve	0.86	0.63; 1.09
7. Putting on a glove	1.00	/
8. Putting on a splint	1.00	/
**Section B**		
1. Difficulty with balance when walking due to your arm	0.53	0.26; 0.81
2. Hold an object still while using unaffected hand	1.00	/
3. Open (affected hand) a previously opened jar	0.79	0.62; 0.96
4. Pick up a glass, bottle, or can	0.89	0.80; 0.97
5. Drink from a cup or mug	0.82	0.65; 0.99
6. Brush your teeth	0.85	0.73; 0.97
7. Tuck in your shirt	0.83	0.65; 1.00
8. Write on paper	0.78	0.63; 0.92
9. Eat with a knife and fork	0.94	0.88; 1.00
10. Dial a number on home phone	0.92	0.86; 0.98
11. Do up buttons on clothing	0.78	0.64; 0.93
12. Comb or brush your hair	0.82	0.74; 1.02
13. Use a key to unlock the door	0.90	0.84; 0.98
**Subscale**	**ICC**_3, 1_	**95% confidence interval (lower; upper)**
Section A	0.87	0.76; 0.93
Section B	0.93	0.87; 0.97

### 3.3. Validity

**Figure 1:**
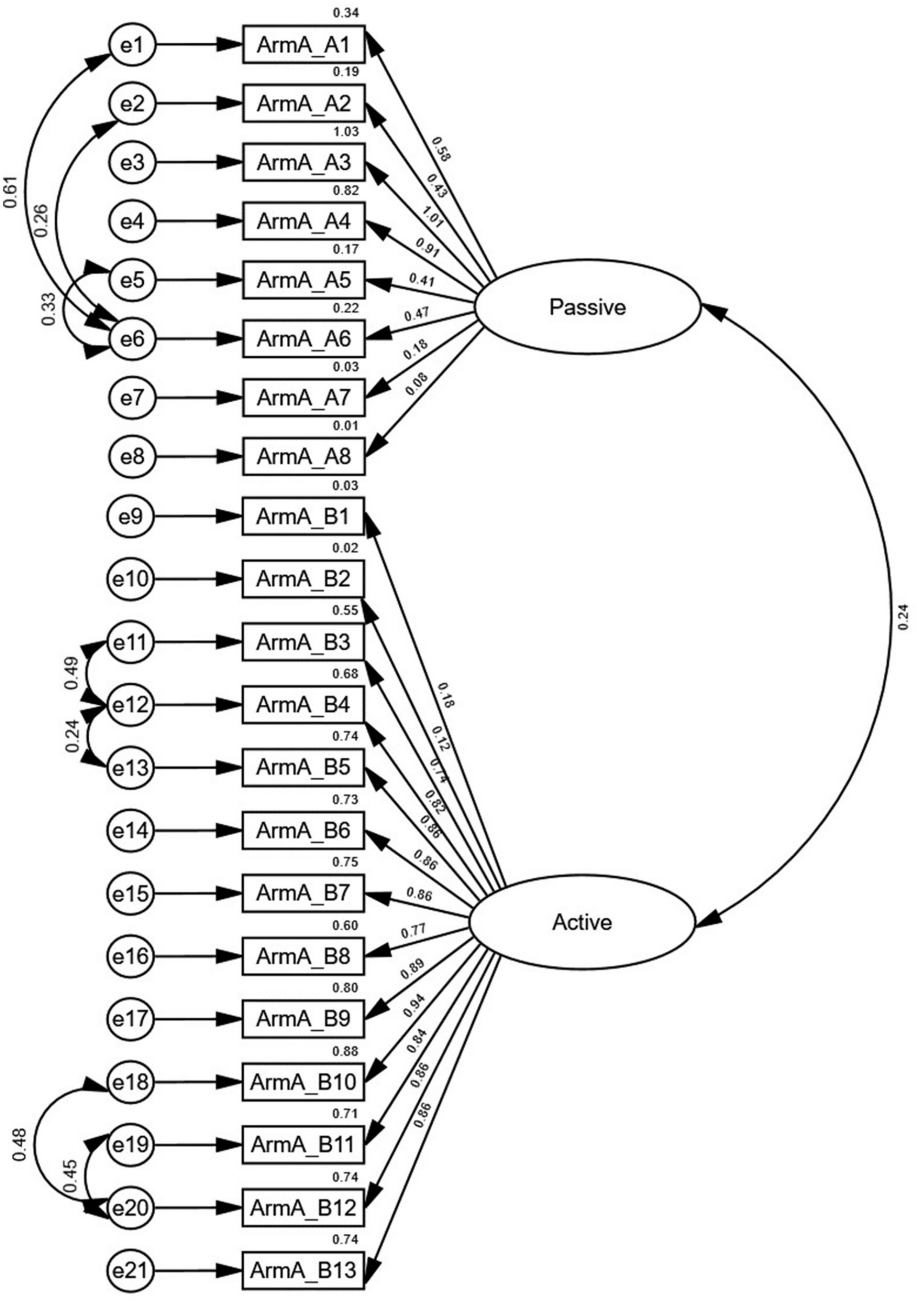
Confirmatory factor analysis of the Chinese version of the Arm Activity Measure.

All items in the ArmA-C obtained an I-CVI value of 1 except items 9 and 12 in section B, which had I-CVI values of 0.4 and 0.8, respectively. The S-CVI value was 0.96. The Cronbach's alpha coefficients for sections A and B were 0.75 and 0.95, respectively (Table 4). The confirmatory factor analysis model of ArmA-C is presented in Figure 1. Although the RMSEA of 0.08 in the model did not reach the criteria, the model displayed an acceptable fit with the CFI of 0.92 and χ^2^/df of 1.74 (*p* < 0.001).

**Table 4:** Internal consistency of the Chinese version of the Arm Activity Measure.

**Item**	**Cronbach's Alpha**	**Corrected item-total correlation**	**Alpha if item deleted**
**Section A**	**0.75**		
1. Cleaning the palm of the hand		0.69	0.69
2. Cutting finger nails		0.43	0.78
3. Cleaning the armpit		0.71	0.68
4. Cleaning the elbow crease		0.61	0.70
5. Positioning arm on a cushion or support in sitting		0.48	0.73
6. Putting arm through a garment sleeve		0.68	0.68
7. Putting on a glove		0.20	0.77
8. Putting on a splint		0.18	0.76
**Section B**	**0.95**		
1. Difficulty with balance when walking due to your arm		0.18	0.96
2. Hold an object still while using unaffected hand		0.14	0.96
3. Open (affected hand) a previously opened jar		0.75	0.95
4. Pick up a glass, bottle, or can		0.83	0.95
5. Drink from a cup or mug		0.86	0.95
6. Brush your teeth		0.83	0.95
7. Tuck in your shirt		0.86	0.95
8. Write on paper		0.76	0.95
9. Eat with a knife and fork		0.86	0.95
10. Dial a number on home phone		0.92	0.94
11. Do up buttons on clothing		0.83	0.95
12. Comb or brush your hair		0.85	0.95
13. Use a key to unlock the door		0.83	0.95

Table 5 summarizes the correlations between ArmA-C scores and other outcome measures. The ArmA-C scores demonstrated significant strong to very strong correlations with the FMA-UE score and WMFT score of the affected side (*r*_*s*_ = −0.40 to −0.81), which supported the hypotheses regarding the construct validity. However, the correlation between the section A of ArmA-C and grip strength was weak (*r*_*s*_ = −0.29) and did not support the hypothesis regarding the construct validity. The ArmA-C scores demonstrated significant weak to strong correlations with the TWT time (*r*_*s*_ = 0.29 to 0.45) and Ox-PAQ-C scores (*r*_*s*_ = 0.20 to 0.51). The correlation between the section B of ArmA-C and TWT time was strong (*r*_*s*_ = 0.45), and that between the section A of ArmA-C and routine activities subscale (*r*_*s*_ = 0.51), and emotional wellbeing subscale of Ox-PAQ-C (*r*_*s*_ = 0.44) were strong, which did not support the hypotheses regarding the construct validity.

**Table 5:** Correlations between the Chinese version of the Arm Activity Measure and other outcome measures.

**Variables**	**Section A**		**Section B**	
	***r**_*s*_*	***p***	***r**_*s*_*	***p***
Fugl-Meyer Assessment for Upper Extremity	−0.42^**^	< 0.001	−0.81^**^	< 0.001
Grip strength (affected side)	−0.29^**^	0.006	−0.59^**^	< 0.001
**Wolf Motor Function Test**				
Affected side	−0.40^**^	< 0.001	−0.79^**^	< 0.001
Unaffected side	−0.09	0.383	−0.20	0.050
Trail Walking Test	0.28^**^	0.005	0.45^**^	< 0.001
**Oxford Participation and Activities Questionnaire**				
Routine activities subscale	0.51^**^	< 0.001	0.37^**^	< 0.001
Social engagement subscale	0.34^**^	< 0.001	0.25^*^	0.013
Emotional wellbeing subscale	0.44^**^	< 0.001	0.20^*^	0.049

* p < 0.05.

** p < 0.01.

## 4. Discussion

This is the first study to translate the ArmA into Chinese and use it to evaluate the active and passive functional upper-limb performance in people with chronic stroke. The ArmA-C demonstrated good test–retest reliability, content validity, and internal consistency. Moreover, the ArmA-C scores had good construct validity by demonstrating acceptable fit to two-factor structure model in the confirmatory factor analysis and significant correlations with the FMA-UE score, grip strength, WMFT scores, TWT completion time, and Ox-PAQ-C scores.

The findings of this study revealed that people with chronic stroke (median scores for sections A and B: 1 and 28.5, respectively) had less difficulty in performing functional tasks with their upper limb than the participants of studies conducted using the original English (median scores for sections A and B: 12 and 48, respectively) (15) and Swedish (median scores for sections A and B: 12 and 46, respectively) versions of the ArmA (17). These discrepancies may be attributed to the different characteristics of the samples. The studies using the English and Swedish versions of the ArmA recruited people with stroke and other neurological conditions who had upper-limb spasticity (e.g., a median Modified Ashworth Scale score of 3 in the Swedish study), which may impair their upper-limb functions. However, our study involved community-dwelling people with chronic stroke only, who were more active and took initiative to participate in their daily activities. They may have developed compensatory strategies to accommodate constraints in the functional tasks. Thus, they may have had better active and passive functions and obtained lower scores in the ArmA-C than the participants in previous studies.

Consistent with previous findings (40), there was no significant difference in ArmA-C scores between participants with and without dominant-side hemiplegia. The possible reason is that people with dominant-side hemiplegia may be more motivated to use their dominant arm because they were not used to using their non-dominant arm before the stroke (40). The propensity to use the dominant arm may produce a “training effect” and support the recovery of the affected arm (40). This may explain why no significant difference in functional performance existed between participants with and without dominant-side hemiplegia.

However, there were significant differences in active and passive functions between stroke participants with mild-to-moderate and severe impairment of the upper extremities, as measured by the FMA-UE. Participants with a greater impairment level obtained significantly higher scores in sections A and B of the ArmA-C. Impairment after stroke may affect the level of spasticity, muscle strength, and joint mobility of the upper limb, which may lead to functional limitations during daily activities (2), resulting in greater difficulty in performing active and passive upper-limb tasks.

Good test–retest reliability was found for sections A and B of the ArmA-C, with similar results to the original English and Swedish versions of the ArmA (15, 17). The item-level agreement was moderate to almost perfect, ranging from 0.53 to 1.00. The ArmA-C had lower item-level test–retest reliability than the original English version of the ArmA (quadratic weighted Kappa coefficients = 0.71–0.94) (15). The possible reason is that a 1-day interval was chosen in the original ArmA, whereas a 1-week interval was used in our study for the evaluation of test–retest reliability. A short test–retest interval may result in higher Kappa coefficient values due to the risk of recall bias.

In contrast to the original English (15) and Swedish versions of the ArmA (17), a floor effect was present in section A of the ArmA-C. Previous studies have shown that the original English ArmA had a ceiling effect in section B (15), but the Swedish ArmA had no ceiling or floor effects in the two subscales (17). One possible reason for the floor effect observed in the ArmA-C was that our participants were community-dwelling people with chronic stroke. They may have developed their own compensatory strategies after their stroke to perform personal care in their daily lives. Thus, they may have perceived less difficulty in performing the upper-limb tasks in section A.

The professional panel supported the content validity of the ArmA-C, with I-CVI values of 0.4 to 1 and an S-CVI value of 0.96. Items 9 and 12 in section B had relatively low I-CVI values of 0.4 and 0.8, respectively, due to the use of Chinese wording. The overall good content validity may be due to the linguistic and contextual equivalence of the ArmA-C and ArmA for the evaluation of arm functions in people with stroke.

The two subscales of the ArmA-C showed good internal consistency. The Cronbach's alpha coefficient was similar between section B of the ArmA-C (Cronbach's alpha = 0.95) and other versions of the ArmA, including the original English version (Cronbach's alpha = 0.96) (15), the Thai version (Cronbach's alpha = 0.88) (16), and the Swedish version (Cronbach's alpha = 0.93) (17). However, the internal consistency of section A of the ArmA-C (Cronbach's alpha = 0.75) was lower than the internal consistency of other versions, which had Cronbach's alpha coefficients of 0.85 to 0.94 (15–17). This discrepancy may be due to the low item–total correlations in items 7 and 8. Eighty-five percent and 97% of participants scored 0 for items 7 and 8, respectively. Some participants had not performed the tasks in items 7 and 8 in section A and they scored 0 for these items according to the original guidelines, which was also interpreted as no difficulty. These misinterpretations may have contributed to the weak correlations between these two items and the subscale, resulting in relatively low internal consistency.

Our study performed confirmatory factor analysis to assess the fit of data in ArmA-C for the two-factor structure of the original English version of the ArmA (“Passive function” and “Active function”). Although the RMSEA in the model did not reach the criteria, this model displayed an acceptable goodness of fit. The possible reason may be different characteristics of the sample in previous study and this study. In the original English version of the ArmA, people with upper-limb paresis due to stroke, brain injuries, and other neurological conditions, were recruited (15), whereas only people with chronic stroke were recruited in this study. Participants with various diagnoses and different signs and symptoms may perceive various levels of difficulty in performing tasks related to personal care and object manipulation. Thus, their perceptions of their functional limitations may be different from the perceptions of people with stroke and other neurological conditions, resulting in a discrepancy in the results. Further studies will be required to investigate the underlying factors influencing the factor structure model of ArmA-C.

The results of the construct validity in this study supported our stated hypotheses and suggested the construct of ArmA-C was similar with FMA-UE and WMFT. Significant strong to very strong correlations were found between the ArmA-C scores and FMA-UE score. Upper-limb impairment after stroke may negatively influence muscle strength, range of motion, and hand dexterity (2, 41). These changes affect functional performance during personal care and object manipulation by the hands of people with stroke, resulting in strong correlations between ArmA-C and FMA-UE scores. The ArmA-C scores also demonstrated significant strong to very strong correlations with the WMFT scores of the affected arm, as the single- or multiple-joint functional movements included in the WMFT are related to difficulty in performing upper limb tasks in the ArmA-C. For example, item 8 (“lift can”) in the WMFT was linked to item 4 (“pick up a glass, bottle, or can”) in section B of the ArmA-C because both of these items assess the ability of cylindrical grasping. Furthermore, item 2 (“forearm to box [side]”) in the WMFT, which requires the participant to place their forearm on a box, is related to item 6 (“putting arm through a garment sleeve”) in section A of the ArmA-C, due to the involvement of shoulder abduction in both items.

Our results also demonstrated that the grip strength of the affected hand was correlated with the ArmA-C scores, which was consistent with the findings of a previous study, illustrating the relationship between grip strength and upper extremity function in people with stroke (*r* = 0.70, *p* < 0.01) (42). Reduced grip strength may decrease arm stability, confine hand usage, and affect upper-limb control and coordination (43). These factors directly influence active and passive functions in the daily lives of people with stroke. However, our stated hypothesis was not supported by the result of construct validity due to weak correlation between the section A of ArmA-C and grip strength. The section A of ArmA-C assesses the ability to care for the affected arm and its items can be performed under the assistance of unaffected arm of people with stroke. Therefore, this may cause weak correlation between the section A of ArmA-C and grip strength of affected hand.

Surprisingly, significant weak to strong correlations were demonstrated between the ArmA-C scores and TWT completion times in this study, which did not support our hypothesis. The TWT examines gait control, which involves the walking performance and cognitive function of people with stroke (30). Previous studies have suggested the important role of arm movements in maintaining coordination between the upper and lower body and gait stability during walking (9, 44) and upper-limb function was related to walking ability in people with stroke (β = 0.11, *p* = 0.047) (8). Another possible explanation for the strong correlation between the section B of ArmA-C scores and TWT completion time is that cognitive functions contribute to the performance of upper-limb tasks in people with stroke (45). Lin et al. found that cognitive impairment explained a substantial proportion of the variance (33%) in functional upper-limb tasks in people with stroke (45). The performance of object manipulation during daily activities also demands cognitive abilities, such as executive function, visual perception, and proprioception. Therefore, both motor and cognitive function assessed in the TWT may be correlated with passive functions, resulting in significant strong correlation the section B of ArmA-C scores and TWT completion time.

Other hypotheses were not supported because the ArmA-C scores also showed significant weak to strong correlations with the Ox-PAQ-C scores, including the routine activities, social engagement, and emotional wellbeing subscales. Upper-limb impairment after stroke may cause limitations in routine activities in people with stroke, including reaching for, grasping, manipulating, transporting, and releasing objects (46). Thus, significant strong correlation was presented between ArmA-C scores and routine activities subscale scores in the Ox-PAQ-C. In addition, reduced upper-limb motor function may also lead to restrictions in social participation by people with stroke due to difficulties in maintaining relationships and transportation (47). The reduced capacity to perform daily tasks and participate in social roles may also affect the emotional and psychological wellbeing of people with stroke (47), resulting in a significant strong correlation between the emotional wellbeing subscale scores in the Ox-PAQ-C and ArmA-C scores.

This study has several limitations that should be noted. First, the study comprised community-dwelling people with chronic stroke who may have better upper-limb motor function than other people with stroke. The findings of this study may not be generalisable to people with stroke with more severe upper-limb motor deficits. Further studies should involve individuals with different levels of post-stroke impairment or different stroke phases to examine the applicability of the ArmA-C. Second, as women had significantly worse functional performance than men after stroke (48), the uneven sex ratio of the participants may have affected the results in this study. Third, the sample size of people with stroke was barely enough to conduct factor analysis. A larger sample size of people with other diagnoses is recommended to further explore the factor structure of the ArmA-C in future studies and provide a more robust conclusion regarding the two-factor structure model. Finally, a limited number of outcome measures were included in the present study. More assessment tools may be necessary in correlation analyses to evaluate the construct validity of ArmA-C scores and in people with stroke.

## 5. Conclusions

The ArmA-C was shown to have good test–retest reliability, content validity, internal consistency, and construct validity in community-dwelling people with chronic stroke. It can be adopted in clinical practice to examine active and passive functions in people with upper-limb impairment. This may facilitate our understanding of their functional performance in real-life contexts.

## Data availability statement

The raw data supporting the conclusions of this article will be made available by the authors, without undue reservation.

## Ethics statement

The studies involving humans were approved by Human Subjects Ethics Committee of The Hong Kong Polytechnic University. The studies were conducted in accordance with the local legislation and institutional requirements. The participants provided their written informed consent to participate in this study.

## Author contributions

NC and SN conceptualized the study and wrote the main manuscript text. Both authors reviewed the manuscript and contributed to manuscript revision, read, and approved the submitted version of the manuscript.
