# Supplementary material for: Psychometric properties of the Chinese version of the Arm Activity Measure in people with chronic stroke
Source: Front Neurol. 2023 Sep 22;14:1248589. doi: 10.3389/fneur.2023.1248589 (PMC10556664; doi:10.3389/fneur.2023.1248589)
Supplement: Supplementary file 1 [file Table_1.DOCX]

Supplementary Material

Psychometric Properties of the Chinese Version of the Arm Activity Measure in People with Chronic Stroke

Nga Huen Chan^1,2^, Shamay S.M. Ng ^1,2,*^

^1^ Department of Rehabilitation Sciences, The Hong Kong Polytechnic University, Hung Hom, Hong Kong SAR, China

^2^ Research Centre for Chinese Medicine Innovation, The Hong Kong Polytechnic University, Hung Hom, Hong Kong SAR, China

*** Correspondence:**Shamay S.M. Ng
[shamay.ng@polyu.edu.hk](mailto:shamay.ng@polyu.edu.hk)

**Supplementary Material**

Supplementary Material 1. The Chinese version of Arm Activity Measure

**《上肢活動量表》填寫指引**

**甲部** 問及(1)您運用健側上肢, (2)您的照顧者, 或(3)您和您的照顧者一起, 如何“照料” 您患側上肢。本部分不會問及您使用患側上肢來完成任何任務。

**乙部** 問及您能使用患側上肢或使用兩側上肢來做甚麼。

就每項列出的活動，請指出(圈出)：

1. 您或您的照顧者在過去7天進行活動時遇到多少困難。如您會進行該活動但在過去7天卻未曾進行（例如剪指甲），請估計其困難程度。

2.如您從不進行該活動，但您估計該活動不涉及您的上肢或您從不用患側上肢進行該活動，給予0分=無困難。

如您未能自行完成問卷，您可以：

- 由照顧者或專業人士協助充當填寫員。
- 由照顧者或專業人士協助去理解和完成每條問題。
- 由照顧者根據您進行活動時所遇到的困難代為完成問卷。

| 請就每項圈出您或您的照顧者在過去7天內進行該活動時遇到的困難程度。 | | | | | | |  |
| --- | --- | --- | --- | --- | --- | --- | --- |
| 活動(患側上肢) | | | **困難程度**  0 = 從沒困難  1 = 輕微困難  2 = 中等困難  3 = 非常困難  4 = 未能做到該活動 | | | |  |
| 甲部 照料您的患側上肢(與進行任務或活動無關) | | | | | | |  |
| 1. 清潔手掌心 | **0** | **1** | | **2** | **3** | **4** | |
| 2. 修剪手指甲 | **0** | **1** | | **2** | **3** | **4** | |
| 3. 清潔腋下 | **0** | **1** | | **2** | **3** | **4** | |
| 4. 清潔手肘皺褶 | **0** | **1** | | **2** | **3** | **4** | |
| 5. 把上肢放在墊子或坐下時承托著（如從沒進行請圈0） | **0** | **1** | | **2** | **3** | **4** | |
| 6. 把上肢穿過衣袖 | **0** | **1** | | **2** | **3** | **4** | |
| 7. 戴上手套（如從沒進行請圈0） | **0** | **1** | | **2** | **3** | **4** | |
| 8. 戴上輔助手托（如從沒進行請圈0） | **0** | **1** | | **2** | **3** | **4** | |
| 乙部 使用患側上肢自行完成任務或活動 | | | | | | |  |
| 1. 因為上肢以致走路時保持平衡有困難 | **0** | **1** | | **2** | **3** | **4** | |
| 2. 使用健側手拿穩物件 | **0** | **1** | | **2** | **3** | **4** | |
| 3. 打開（患側手）一個已開啟的瓶 | **0** | **1** | | **2** | **3** | **4** | |
| 4. 拿起一個玻璃杯，樽或罐 | **0** | **1** | | **2** | **3** | **4** | |
| 5. 使用有柄茶杯或有柄大杯飲水 | **0** | **1** | | **2** | **3** | **4** | |
| 6. 刷牙 | **0** | **1** | | **2** | **3** | **4** | |
| 7. 把衣服紮進褲子 | **0** | **1** | | **2** | **3** | **4** | |
| 8. 在紙上寫字 | **0** | **1** | | **2** | **3** | **4** | |
| 9. 使用餐具進食 | **0** | **1** | | **2** | **3** | **4** | |
| 10.撥打家居電話 | **0** | **1** | | **2** | **3** | **4** | |
| 11.扣好衣服的鈕扣 | **0** | **1** | | **2** | **3** | **4** | |
| 12.梳頭 | **0** | **1** | | **2** | **3** | **4** | |
| 13.用鑰匙開門 | **0** | **1** | | **2** | **3** | **4** | |

總分 甲部 □□

乙部 □□

甲部和乙部的總分分別是該部份的總和。

*兩部份不應合併計算。*
